# Heat Wave Adaptations: Unraveling the Competitive Dynamics Between Invasive *Wedelia trilobata* and Native *Wedelia chinensis*

**DOI:** 10.3390/plants13243480

**Published:** 2024-12-12

**Authors:** Haochen Yu, Cheng Han, Guangqian Ren, Xuanwen Wu, Shanshan Qi, Bin Yang, Miaomiao Cui, Xue Fan, Zhaoqi Zhu, Zhicong Dai, Daolin Du

**Affiliations:** 1 School of Emergency Management, Jiangsu University, Zhenjiang 212013, China; 2 School of Environment and Safety Engineering, Jiangsu University, Zhenjiang 212013, China; 3 Shanghai Meteorological Service Centre, Shanghai 200030, China; 4 Jiangsu Province Engineering Research Center of Green Technology and Contingency Management for Emerging Pollutants, Jiangsu University, Zhenjiang 212013, China; 5 School of Tourism Management, Jiangsu College of Tourism, Yangzhou 225000, China; 6 School of Agricultural Engineering, Jiangsu University, Zhenjiang 212013, China; 7 College of Life Sciences, Shenyang Normal University, Shenyang 110034, China; 8 School of Environmental Engineering, Nanjing Institute of Technology, Nanjing 211167, China; 9 Institute of Environment and Ecology, School of Environment and Safety Engineering, Jiangsu University, Zhenjiang 212013, China; 10 Jiangsu Collaborative Innovation Center of Technology and Material of Water Treatment, Suzhou University of Science and Technology, Suzhou 215009, China; 11 Jingjiang College, Jiangsu University, Zhenjiang 212013, China

**Keywords:** plant invasion, adaptive strategies, physiological response, global warming, thermal resilience

## Abstract

Heat waves (HW) are projected to become more frequent and intense with climate change, potentially enhancing the invasiveness of certain plant species. This study aims to compare the physiological and photosynthetic responses of the invasive *Wedelia trilobata* and its native congener *Wedelia chinensis* under simulated heat wave conditions (40.1 °C, derived from local historical data). Results show that *W. trilobata* maintained higher photosynthetic efficiency, water-use efficiency (WUE), and total biomass under HW, suggesting that its ability to optimize above-ground growth contributes to its success in heat-prone environments. In contrast, *W. chinensis* focused more on root development and antioxidant protection, exhibiting a decrease in total biomass under heat wave conditions. These results indicate that *W. trilobata* employs a more effective strategy to cope with heat stress, likely enhancing its competitive advantage in regions affected by heat waves. This study highlights the importance of understanding species-specific responses to extreme climate events and underscores the potential for heat waves to drive ecological shifts, favoring invasive species with higher phenotypic plasticity.

## 1. Introduction

An unprecedented invasion of alien plant species to areas outside their native ranges has being caused by the expansion of transportation systems and the increasing movement of individuals and products around the world [1,2,3]. Given the robust evidence that many invasive alien plants have major negative impacts on human health [4], economic activities [5], and biodiversity [6], understanding the role the invasive plants play in biological invasions is crucial to secure sustainable ecological development [7].

Significant increases in extreme climatic events over the past ten years have revealed a concerning gap in the scientific literature regarding their ecological effects. Scientists have placed an excessive emphasis on mean change rather than extremes, even though it is the extremes that cause world-wide effects such as heat waves (HW) [8]. Climate change is anticipated to cause more frequent and intense weather-related events such as HW [9]. Although there is no universal definition of HW, such extreme events linked to exceedingly high temperature and short time period have been shown to have substantial impacts on human mortality, local economies, and ecosystems [10,11]. Consequently, growing season temperatures and their variance will increase, changing the phenology, physiological performance, and productivity of invasive plants [12].

Interest in invasive plant heat stress response has never been greater, mainly due to concerns regarding how managed and natural ecosystems will be affected by heat stress. HW affects a variety of physiological and biochemical processes in plants. These include cell growth, division and differentiation, photosynthesis, respiration, water potential, increase in transpiration, and nutrient uptake and transport [8]. “Priming”, which subjects plants to a simulated HW, has been shown to increase their ability to withstand subsequent episodes of heat stress [13]. This thermotolerance is typically observed through indicators like electrolyte leakage, the heat resistance of photosystem II’s maximum photochemical efficiency (Fv/Fm), and the viability of seeds or seedlings [14,15]. Warming was found to decrease the germination of invasive *Solidago canadensis*, but it advanced the seed germination time and increased germination rate of native *S*. *canadensis* [16]. Under intense heat, *Spartina patens* showed greater photosynthetic efficiency (α) than the native *S. maritima*. This decrease in photosynthetic efficiency was mainly due to reduced connectivity among PSII (photosystem II) antennae, along with considerable damage to the PSII oxygen-evolving complex [17]. Elevated temperature was likely to increase the effectiveness of *Alternanthera philoxeroides*’s interspecific competition with the native co-occurring species *Digitaria sanguinalis* [18]. Warming reduced nitrogen levels in *Alternanthera philoxeroides* and heightened total flavonoid and phenol content in its native congener *Alternanthera sessilis* [19]. At the cellular level, HW results in excessive production of reactive oxygen species (ROS), which contributes to transduction of the heat signal, leading to the regulation of expression of heat shock protein (HSP) genes involved in thermotolerance [20]. HW triggers protein denaturation and deformation, which creates proteotoxic stress and disrupts membrane stability and cytoskeleton integrity, leading to collapse of cellular structure [21].

The physiological and photosynthetic responses of invasive *Wedelia trilobata* (*W. trilobata*) and native *Wedelia chinensis* (*W. chinensis*) to HW treatments were investigated. *Wedelia trilobata* (L.) Hitchc. (Asteraceae), native to the tropics of South America, is one of the malignant invasive weeds in the world, and it is widely distributed in southern China [22,23]. This plant reproduces primarily asexually, with its stem segments exhibiting a high level of plasticity. This adaptability enables it to spread quickly through various propagation methods, including cutting, stripping, and even soilless cultivation [24]. Introduced to China in the 1970s for ornamental purposes, this plant tends to smother other vegetation, forming dense covers that hinder the growth and regeneration of different species, thus emerging as a significant invasive weed in southern China [25]. *Wedelia trilobata* was found to be only infrequently attacked by herbivores or pathogens in invaded habitats [26]. *Wedelia chinensis* (Osbeck.) Merr. (Asteraceae) is the native congener of *W. trilobata* in China [27]. Both species exhibit comparable morphological and life historical characteristics. Unlike *W. trilobata*, *W. chinensis* exhibits a slower growth rate and, within China, it has not been identified as detrimental to indigenous flora or ecosystems. This plant is valued for its medicinal uses and contains bioactive compounds such as flavonoids, diterpenes, triterpene saponins, and phytosteroids [28]. It also has antioxidant [29], anti-inflammatory and antimicrobial [30], and anticancer effects [31]. Under high-temperature stress, *W. trilobata* shows less inhibition of relative growth rate (RGR) and biomass production, maintaining higher photosystem II (PSII) activity and net photosynthetic rate (Pn) than *W. chinensis* [27]. Additionally, *W. trilobata* demonstrates superior water retention, with a slower water loss rate in detached leaves compared to *W. chinensis* [32]. The species also shows enhanced antioxidant activity [33] and gene expression under competitive conditions including drought stress [34], as well as better physiological responses under low nitrogen [23] or low temperature and light conditions [35]. Furthermore, gibberellins (GAs) accelerate its clonal growth more effectively than in *W. chinensis*, suggesting a strong hormonal influence on its invasiveness [36]. These traits, combined with an efficient antioxidant defense system [33], enable *W. trilobata* to thrive in competitive and stress-prone environments.

The following hypotheses were tested in this study: (1) Heat waves will limit the phenotypic growth of both *Wedelia trilobata* (L.) Hitchc. and *Wedelia chinensis* (Osbeck.) Merr., but the extent of growth limitation will differ, with the invasive species potentially exhibiting greater tolerance. (2) Are there differences in photosynthetic adaptation strategies of *W. trilobata* and *W. chinensis*, and if so, what are the different adaptation mechanisms facing heat waves?

## 2. Result and Discussion

### 2.1. Divergent Physiological Adaptations to HW in Wedelia trilobata and Wedelia chinensis

For *Wedelia trilobata*, HW significantly impacted leaf mass (*p* < 0.001) and root length (*p* < 0.05) (Table 1). Culture conditions had a significant effect on leaf surface area (*p* < 0.01) and plant height (*p* < 0.05). Additionally, the interaction between heat wave treatment and culture conditions significantly influenced total biomass (*p* < 0.05) and leaf mass (*p* < 0.01), suggesting that the effects of heat wave treatment on these parameters were moderated by the culture conditions. In *W. chinensis*, HW led to significant changes in leaf mass (*p* < 0.01) and leaf surface area (*p* < 0.05). Culture conditions had a significant effect on several physiological parameters in *W. chinensis*’s leaf mass (*p* < 0.001), leaf surface area (*p* < 0.001), plant height (*p* < 0.001), and root length (*p* < 0.05).

**Figure 1:**
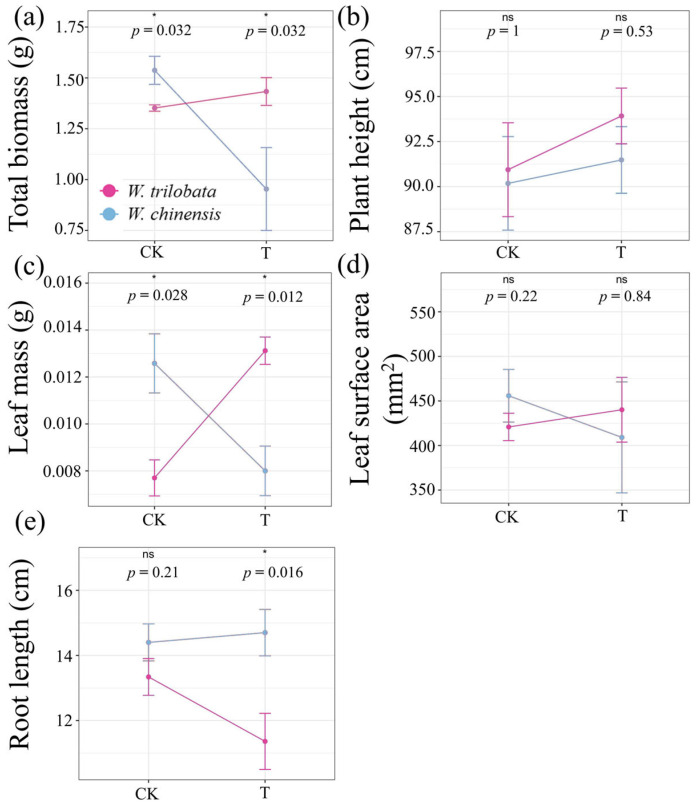
Comparative response of *W. trilobata* and *W. chinensis* to control (CK) and heat wave (T) in mixed culture conditions. Graphs (**a**–**e**) display the mean values and standard errors for plant height, total biomass, root length, leaf mass, and leaf surface area, respectively. Statistically significant differences are indicated as follows: ‘ns’ denotes no statistically significant difference, while * indicates a statistically significant difference at *p* < 0.05.

Under heat wave and mixed culture conditions, *W. trilobata* shows an increase in total biomass, while *W. chinensis* experiences a decrease (*p* < 0.01) (Figure 1a). Both species exhibit an increase in plant height under HW; however, the increase is more pronounced in *W. trilobata* compared to *W. chinensis* (Figure 1b). For leaf mass, *W. trilobata* increases its leaf mass under heat wave conditions, surpassing *W. chinensis*, which instead shows a decrease (*p* < 0.05) (Figure 1c). Leaf surface area remains relatively stable for both species across control and heat wave conditions, indicating no significant changes (Figure 1d). Regarding root length, *W. trilobata* shows a decrease, while *W. chinensis* slightly increases its root length under heat wave conditions (*p* < 0.05) (Figure 1e). These findings suggest that *W. trilobata* and *W. chinensis* employ different adaptive strategies under HW, with *W. trilobata* favoring above-ground growth and *W. chinensis* potentially investing more in root development. This supports our first hypothesis that heat wave conditions limit phenotypic growth in both *W. trilobata* and *W. chinensis*, with *W. trilobata* demonstrating greater tolerance.

Phenotypic plasticity is a key mechanism that enables plants to become invasive species under stress [37], as it serves as the primary method for adapting to variations in environmental factors, such as temperature [38]. High temperatures affect various physiological processes, but the extent of inhibition varies greatly between plant species, significantly influencing plant competition. After brief exposure to heat wave, the growth of two *Wedelia* species was restricted to varying extents.

Our study highlights the contrasting adaptive strategies of *W. trilobata* and *W. chinensis* under heat wave conditions. Our results align with the concept that phenotypic plasticity, particularly in terms of resource allocation among organs, plays a crucial role in plant adaptability to extreme temperature conditions [39]. In the case of *W. trilobata*, its robust above-ground response under HW, marked by increases in total biomass, leaf mass, leaf surface area, and plant height, suggests a strategic allocation of resources toward above-ground growth [40]. This shift likely enhances photosynthetic capacity, supporting the plant’s overall resilience to elevated temperatures. Although the increases in leaf surface area and plant height were not statistically significant, the trend further underscores the plant’s focus on maximizing photosynthetic potential under HW. This resource reallocation supports previous findings that species exhibiting greater phenotypic plasticity, such as *W. trilobata*, can better adapt to varying environmental conditions [24]. The ability of *W. trilobata* to increase specific leaf area (SLA) and plant height under warming conditions reflects its inherent adaptability [41]. This phenotypic flexibility likely contributes to its capacity to tolerate higher temperatures and may explain its broader distribution in heat-prone regions. These adaptive traits highlight the importance of resource trade-offs in allowing species to thrive under fluctuating environmental conditions, facilitating their expansion across diverse habitats.

In contrast, *W. chinensis* shows a decrease in total biomass and a reduction in leaf mass, while exhibiting a slight increase in root length. Our results are consistent with previous studies showing that *W. chinensis* experiences greater inhibition in total biomass compared to *W. trilobata* under heat stress [27]. This indicates that *W. chinensis* may prioritize root development under HW to improve water uptake. These differences suggest that *W. trilobata* and *W. chinensis* employ distinct physiological strategies to cope with HW, with the former focusing on above-ground growth and the latter investing more in below-ground structures.

### 2.2. Heat Wave Effects on Photosynthesis Traits in Wedelia Species

For *W. trilobata*, HW significantly affected photosynthetic efficiency (Fv/Fm), net photosynthesis rate (Pn), transpiration rate (Tr), CO_2_ assimilation rate, and water-use efficiency (WUE), with *p* < 0.001 for all these parameters (Table 2). Chlorophyll content and leaf nitrogen were also significantly influenced by the interaction between heat wave treatment and culture conditions (T × C) (*p* < 0.001 for both), highlighting the importance of environmental context in determining these traits. Culture conditions had a significant impact on flavonol content (*p* < 0.01), transpiration rate (*p* < 0.001), and WUE (*p* < 0.01), but did not significantly affect chlorophyll content or anthocyanin levels.

In *W. chinensis*, heat wave treatment significantly affected chlorophyll content, leaf nitrogen, flavonol content, anthocyanin levels (all *p* < 0.01), Fv/Fm, Pn, and WUE (all *p* < 0.001). Culture conditions also influenced chlorophyll (*p* < 0.01), leaf nitrogen (*p* < 0.05), flavonol content (*p* < 0.01), and anthocyanin levels (*p* < 0.05). The interaction between heat wave treatment and culture conditions had significant effects on chlorophyll content, leaf nitrogen, anthocyanin levels, and Pn (all *p* < 0.05), as well as WUE (*p* < 0.001), underscoring the combined impact of these factors on physiological responses.

**Figure 2:**
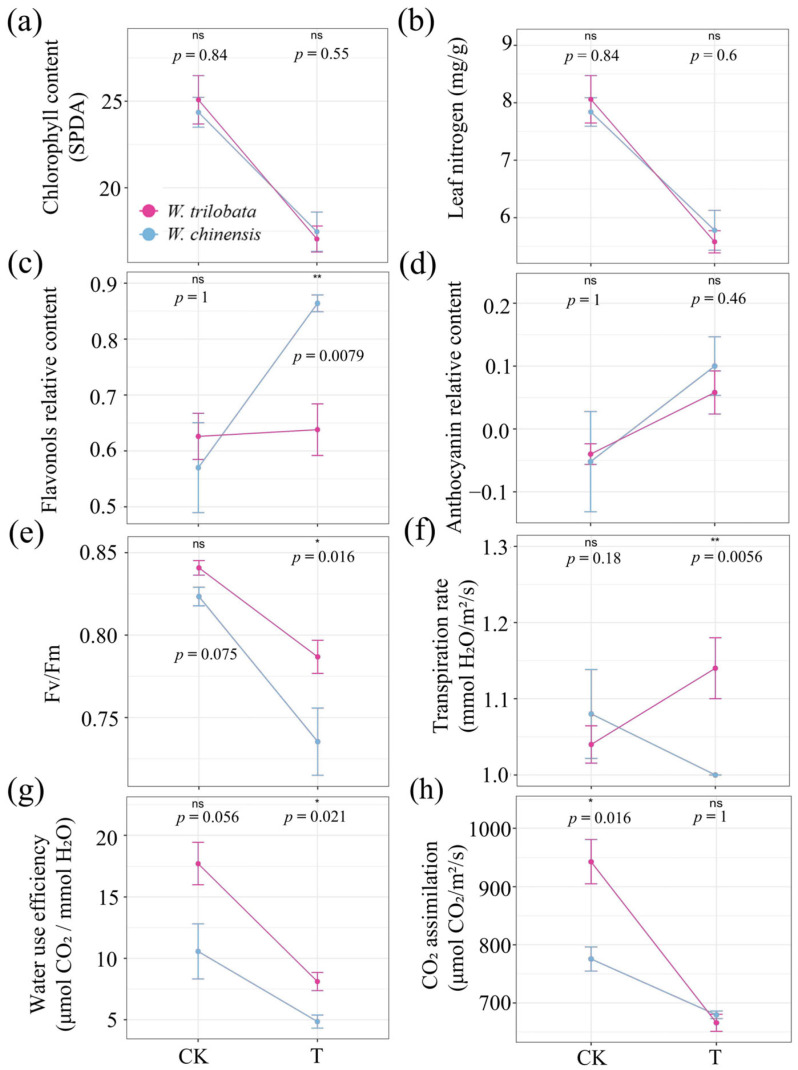
Comparative response of *W. trilobata* and *W. chinensis* to control (CK) and heat wave (T) in mixed culture conditions. Graphs (**a**–**h**) display the mean values and standard errors for chlorophyll content (**a**), leaf nitrogen content (**b**), flavonoid content (**c**), anthocyanin content (**d**), Fv/Fm (**e**), transpiration rate (**f**), water use efficiency (**g**), and CO_2_ assimilation rate (**h**), respectively. Statistically significant differences are indicated as follows: ‘ns’ denotes no statistically significant difference, while * and ** indicate a statistically significant difference at *p* < 0.05 and *p* < 0.01, respectively.

Both species exhibit a decrease in chlorophyll and leaf nitrogen content (Figure 2a,b). Under heat wave and mixed culture conditions, *W. chinensis* shows a significantly higher increase in flavonoid content compared to *W. trilobata* (*p* < 0.01) (Figure 2c), indicating a stronger protective response. Both species increase anthocyanin content under HW, with *W. chinensis* showing a slightly greater increase (Figure 2d). *Wedelia trilobata* maintains higher Fv/Fm under heat wave conditions (*p* < 0.05) (Figure 2e), and shows a greater increase in transpiration rate (Tr) (*p* < 0.01) (Figure 2f). WUE decreases more in *W. trilobata* than in *W. chinensis* under heat wave conditions (*p* < 0.05) (Figure 2g), but it still remains higher in *W. trilobata* compared to *W. chinensis*. Additionally, *W. trilobata* experiences a greater decrease in CO_2_ assimilation rate compared to *W. chinensis* (Figure 2h). Overall, these results highlight distinct adaptive strategies between the two species, with *W. chinensis* potentially focusing on enhancing protective compounds and maintaining CO_2_ assimilation, while *W. trilobata* appears to prioritize increased transpiration and maintaining photosynthetic efficiency.

Photosynthesis is highly sensitive to high-temperature stress, making it a critical process that influences plant metabolism and growth. The primary components affected by heat stress are the photosystem II (PSII) reaction center [42] and CO_2_ assimilation [43], which are particularly vulnerable under heat wave. Exposure to heat wave treatment led to significant alterations in PSII function in both *Wedelia* species. A clear declining trend in Fv/Fm was evident in both species. Changes in Fv/Fm is recognized as reliable diagnostic indicator of photoinhibition [44]. In our results, *W. trilobata* maintained relatively higher PSII activity under high-temperature conditions compared to *W. chinensis*, which is consistent with the findings of Song et al. [27]. The net CO_2_ assimilation in *W. trilobata* strongly declined under heat wave conditions, which may be due to the higher respiration rates of this invasive plant under such extreme temperatures. Elevated respiration rates during heat waves can lead to increased carbon loss, outweighing the carbon gain from photosynthesis, thus contributing to the observed decline in CO_2_ assimilation in our results.

The anthocyanin contents in the leaves of the two species both increased under HW. The anthocyanin content of *W. chinensis* had a higher increase rate than *W. trilobata*. The production of anthocyanins can partially absorb external light energy, helping to reduce the buildup of excess light energy [45]. Our results suggest that the leaves of *W. chinensis* exhibited a stronger screening effect against external light energy during a heat wave, leading to a more effective reduction in the accumulation of excess light energy. Similarly, our results also showed that the increase in flavonoids in the leaves of *W. trilobata*, a potent non-enzymatic antioxidant in plants, was less than that in the native plant, indicating that *W. chinensis* exhibited a stronger antioxidant capacity in the leaves, which is consistent with the findings of Cai et al. under thermal stress [24]. WUE decreases more in *W. trilobata* than in *W. chinensis* under heat wave conditions (Figure 2g); however, WUE remains higher in *W. trilobata* compared to *W. chinensis* after HW. According to Wu et al., this characteristic might confer drought tolerance to *W. trilobata* during heat waves, contributing to its success as an invader [46].

Our results validate our second hypothesis that *W. trilobata* and *W. chinensis* exhibit different photosynthetic adaptation strategies under heat wave conditions. Specifically, *W. trilobata*’s success in invading under heat wave conditions is likely due to its higher WUE, Tr and greater PSII activity, allowing it to maintain photosynthesis and growth under stress. In contrast, *W. chinensis* effectively resists invasion through stronger light protection, with higher anthocyanin production and antioxidant capacity, which helps it manage light and oxidative stress during heat waves.

### 2.3. Calculation-Driven Insights: HW as a Catalyst for W. trilobata’s Invasive Advantage

**Figure 3:**
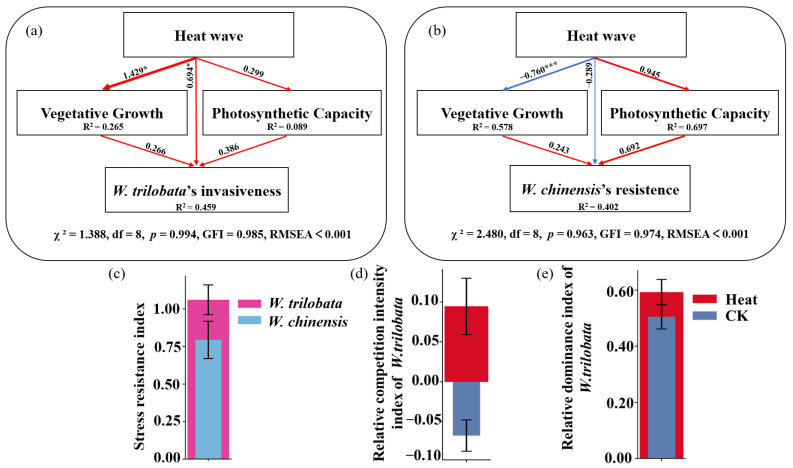
Path analyses and stress response indices of *W. trilobata* and *W. chinensis* under HW conditions. Panels (**a**,**b**) show path analyses for *W. trilobata* and *W. chinensis*, respectively, illustrating the relationships between HW, vegetative growth, photosynthetic capacity, and either invasiveness (*W. trilobata*) or resistance (*W. chinensis*). Statistically significant pathways are indicated by * and ***, with *p* < 0.05 and *p* < 0.001, while R^2^ values represent the proportion of variance explained by the model. Panel (**c**) displays the stress resistance index for both species under HW conditions. Panel (**d**) shows the relative competition intensity index (RCI) for *W. trilobata* against *W. chinensis* under control and HW. Panel (**e**) illustrates the relative dominance index (RDI) of *W. trilobata* against *W. chinensis* under control and HW.

In *W. trilobata* (Figure 3a), heat wave (HW) significantly promoted vegetative growth with a standardized path coefficient of 1.429 (*p* < 0.05), but had a smaller direct effect on photosynthetic capacity (path coefficient = 0.299). Both vegetative growth and photosynthetic capacity positively influenced invasiveness, with path coefficients of 0.266 and 0.386, respectively. The direct path from HW to invasiveness was significant, with a path coefficient of 0.694 (*p* < 0.05). The overall model for *W. trilobata* explained 45.9% of the variance in invasiveness (R^2^ = 0.459), indicating that HW and its effects on vegetative growth and photosynthetic capacity are important drivers of the species’ invasive potential.

In contrast, *W. chinensis* (Figure 3b) exhibited a distinct response under HW. HW significantly reduced vegetative growth (path coefficient = −0.760, *p* < 0.001) but positively affected photosynthetic capacity (path coefficient = 0.945). Vegetative growth had a weak influence on resistance (path coefficient = 0.243), while photosynthetic capacity contributed positively to resistance (path coefficient = 0.692). The negative path coefficient (−0.289) indicates that heat waves directly reduce the resistance of *W. chinensis*. This suggests that *W. chinensis* may be somewhat vulnerable to heat waves, and its ability to resist environmental stressors might be weakened under such conditions. The model for *W. chinensis* accounted for 40.2% of the variance in resistance (R^2^ = 0.402).

*Wedelia trilobata* consistently exhibited higher stress resistance index (SRI) compared to *W. chinensis* (Figure 3c), indicating its greater resilience to HW, potentially due to its enhanced above-ground growth, higher WUE, and greater PSII activity. The relative competition intensity index (RCI) shows that *W. trilobata* demonstrated a competitive advantage over *W. chinensis* under HW, consistent with path analysis results indicating positive effects of HW on *W. trilobata*’s growth and photosynthesis (Figure 3d). Lastly, the relative dominance index (RDI) shows that *W. trilobata* exhibited higher dominance under HW relative to the control condition, suggesting that elevated temperatures enhance its competitive dominance and invasive potential in heat-stressed environments (Figure 3e).

*W. trilobata* not only benefits from HW through enhanced growth and photosynthesis but also shows a direct pathway from HW to invasiveness, suggesting adaptive mechanisms triggered by HW that facilitate its spread. This indicates a potentially greater resilience to HW, allowing *W. trilobata* to thrive and expand even under unfavorable conditions. In contrast, *W. chinensis* appears more vulnerable to HW, experiencing directly negative impacts on both growth and resistance. While *W. chinensis* can maintain photosynthetic capacity, this alone may not suffice to counteract its overall reduction in resilience, making it less competitive and more susceptible to displacement by invasive species under climate-induced HW. The SRI and RCI index indicate that HW did not have a negative impact on *W. trilobata*, but instead enhanced its competitive ability. RDI analysis shows that the relative dominance of *W. trilobata* increased under HW, suggesting a strong invasive potential, especially in environments affected by HW.

Consequently, *W. trilobata* is better positioned to capitalize on HW to enhance its invasiveness, whereas *W. chinensis* may experience suppressed growth and reduced resistance. This dynamic could lead to *W. trilobata*’s increased dominance over *W. chinensis* in HW-affected areas, promoting the establishment and spread of invasive species at the expense of native species. A recent study revealed that arbuscular mycorrhizal fungi (AMF) could enhance *W. trilobata*’s capacity to adapt to abiotic stresses by modulating its metabolic profile. AMF were shown to elevate levels of key metabolites, such as amino acids, organic acids, flavonoids, and plant hormones like L-proline, L-phenylalanine, and abscisic acid [47]. The accumulation of these metabolites may strengthen *W. trilobata*’s ability to cope with HW. However, the allelopathic properties of *W. chinensis* provide a potential avenue for managing *W. trilobata*. The phytotoxic compounds vanillic acid and gallic acid, identified in *W. chinensis*, have shown significant inhibitory effects on plant growth [48]. These compounds could be leveraged as natural bioherbicides or integrated into restoration strategies to suppress *W. trilobata*. Scientists have also identified limited light availability and the accumulation of its own litter as major factors restricting *W. trilobata* seedling emergence in the field [49]. Preventing seed production is crucial to controlling its spread, as eliminating these natural constraints could facilitate the establishment of new populations. These strategies offer eco-friendly solutions to suppress *W. trilobata* while enhancing the resilience of native ecosystems.

## 3. Conclusions

According to the statistics of extremes, “However big floods get, there will always be a greater one coming”; the same could be valid for HW. By employing calculated temperatures, we have demonstrated that 40.1 °C, derived from local historical temperature data, effectively simulates real-world heat wave conditions. Our study reveals that *W. trilobata* leverages a robust above-ground growth strategy, maintaining higher WUE and photosynthetic stability even under intense heat stress. These traits likely underpin its invasive success in warming climates, allowing it to dominate in regions susceptible to heat waves. In contrast, the native *W. chinensis* adopts a conservative strategy focused on root development and increased antioxidant protection, allowing it to withstand heat stress through enhanced water uptake and reduced oxidative damage. This study highlights the critical role of heat waves as selective forces that exacerbate competitive disparities between invasive and native species, with *W. trilobata*’s adaptive plasticity potentially leading to its dominance in heat-prone regions. As global temperatures rise, invasive species like *W. trilobata* may gain a competitive edge, posing challenges for biodiversity conservation. Future research should explore the molecular mechanisms of heat tolerance and investigate the long-term ecological impacts of heat waves on plant communities, while management strategies must account for the intensifying effects of extreme climate events to mitigate the ecological risks of invasive species expansion.

## 4. Materials and Methods

For this study, *Wedelia trilobata* and *W. chinensis* plants were randomly collected from an invaded habitat in Guangdong Province, Jieyang City (23°29′ N and 116°16′ E), and propagated in a greenhouse (32°12′ N, 119°30′ E) at Jiangsu University, Zhenjiang, China. The ramets of these two plant species were cut in two nodes and grown in the greenhouse till to two leaves were opened [23]. For roots and germination, the stem segments were then soaked in 0.1 times Hoagland nutrient solution with a randomized complete block design in the greenhouse of Jiangsu University [50]; the temperature was 25 °C, the humidity was 60%, and the light cycle was 14 h/10 h (day/night). After 14 days, the segments were transplanted in plastic pots.

### 4.1. Experimental Design

The experiment was conducted between July and October 2023. *Wedelia trilobata* and *W. chinensis* segments were placed in square pots (10 cm × 10 cm × 8.5 cm) filled with nutrient soil. We allocated 10 pots to three cultures: native species monoculture (two *W. chinensis* per pot), native and invasive in mixed culture (one *W. trilobata* and one *W. chinensis* per pot), and invasive monoculture (two *W. trilobata* per pot). There were a total of 60 plants in 30 pots. Plants were cultivated in a growth chamber (JNR-508; Ningbo Jiangnan Instrument Factory, Ningbo, China) at 27/25 °C(day/night), 60%/80% (day/night) relative humidity, 14 h photoperiod, and average lumens of 4500 (lx). For the HW treatment, the temperature was set to 40.1 °C for three consecutive days, while the relative humidity and lumen density remained the same.

### 4.2. Determination of Local HW Temperature

Heat waves are described as extremely high temperatures that continue for several days. There are 12 different HW commonly used in the literature [11,51,52,53,54,55]. HW have been characterized by a variety of temperature measurements, including daily maximum apparent temperature [56], daily mean surface air temperature [51], daily maximum surface air temperature [57], and daily minimum surface air temperature [58]. Due to the devastating effects of excessive temperatures, daily maximum surface air temperature was chosen following that of several scientific studies [53,59,60]. While heat wave temperatures have been extensively studied and reviewed, most of the plant experiments still use absolute criterion that designate a specific temperature value, for example, the 35 °C recommended by the China Meteorological Administration (CMA) [61], or the 32 °C recommended by the WMO [62], etc. Scientists suggested that fixed absolute thresholds need to be replaced with locally determined thresholds in the form of high quantiles of the local temperatures to increase applicability across various climatic zones [63].

Here, HW is defined as a period of three consecutive days during which the daily maximum surface temperature is higher than its corresponding historical 90th percentile threshold [64]. By comparing the 15-day temperature samples around a calendar day (i.e., 7 days before and after the calendar day) over a period of 27 years (1996–2022), the 90th percentile threshold was then determined.

### 4.3. Data Collection

Freshly gathered leaves, stems, and roots were rinsed with purified water before being subjected to a drying process. Initially, the samples underwent heating at a temperature of 105 °C for 10 min, succeeded by a prolonged drying phase at 65 °C spanning 72 h [50]. The aggregate of the weight of the dried leaves, stems, and roots constituted the total biomass, measured using an analytical balance (BAS124S, Sartorius, Gottingen, Germany). The net photosynthesis rate (Pn) for each leaf was gauged using a FS-3080H Plant photosynthesis meter (Fansheng Technology, Shijiazhuang, China). To assess the photosynthetic efficiency of each plant, we examined the chlorophyll fluorescence in leaves with a PAR-FluorPen FP 110/D portable fluorometer (PSI, Brno, Czech Republic). For the chlorophyll fluorescence measurements, leaf specimens were prepared by affixing a dark adaptation clip for 15 min. Subsequently, the instrument was deployed to ascertain the initial fluorescence (Fo) and peak fluorescence intensity (Fm) through the OJIP analysis mode. Fv/Fm, where Fv equals Fm minus Fo. The leaf’s relative chlorophyll content was quantified utilizing a SPAD-502 PLUS chlorophyll meter (Konica Minolta, Tokyo, Japan). Moreover, the area of each leaf was quantified via a leaf area meter (YMJ-CH, Tuopuyunnong, Hangzhou, China), and the fresh mass was ascertained with an analytical scale (BAS124S, Sartorius, Gottingen, Germany).

### 4.4. Statistical Analysis

Initial data inspection through the application of Levene’s and Shapiro-Wilks tests indicated violations of the assumptions for normality and homogeneity of variances required for analysis of variance (ANOVA). Consequently, to accommodate the non-parametric nature of factorial ANOVA under these conditions, we employed aligned rank transformation (ART) [15,65]. Structural Equation Modeling (SEM) was employed to investigate the relationships among plant growth and physiological response variables under HW conditions. The analysis was conducted using the lavaan [66] package in R. Data were standardized prior to model fitting.

To assess the degree of resistance exhibited by a given plant species to HW, the stress resistance index [67] (SRI) was calculated according to the following formula:

(1) $$SRI=1-\frac{B_{ns}-B_{s}}{B_{ns}}$$

where B_ns_ and B_s_ represent the total biomass of the plant species i under the condition with stress and the condition without stress, respectively. Plant species with a higher value of SRI demonstrate a greater degree of stress resistance compared to those with a lower value of SRI.

To assess the growth competitiveness of invasive plant species, the relative competition intensity index (RCI) [68] was calculated according to the following formula:

(2) $$RCI=(B_{coi}-B_{mi})/(B_{coi}+B_{mi})$$

RCI represents the relative competition intensity index of species i against species j. B_coi_ is the biomass per plant of species i in the mixed planting with species j, and B_mi_ is the biomass per plant of species i in monoculture. If RCI is greater than 0, it indicates that the relative competition intensity of species i is greater than that of species j. If RCI is less than 0, it indicates that the relative competition intensity of species i is less than that of species j. If RCI equals 0, it indicates that the relative competition intensity of species i is equal to that of species j.

To assess the degree of dominance exhibited by a given plant species, the relative dominance index (RDI) [69] was calculated according to the following formula:

(3) $${RDI}_{ij}=B_{coi}/B_{coij}$$

B_coij_ denotes the total biomass of both species when species i and species j are co-planted. B_coi_ indicates the biomass of species i when co-planted with species j. If RDI > 0.5, it suggests that the relative competitiveness of species i is greater than that of species j. If RDI < 0.5, it indicates that the relative competitiveness of species i is less than that of species j. If RDI = 0.5, it implies that the relative competitiveness of species i is equal to that of species.

## Figures and Tables

**Table 1:** Response of *Wedelia trilobata* and *W. chinensis* to heat wave treatment and culture conditions. The table summarizes the effects of heat wave treatment (T), culture (C), and their interaction (T × C) on five physiological parameters: total biomass, leaf mass, leaf surface area, plant height, and root length.

			*W. trilobata*				*W. chinensis*			
Source	Total Biomass	Leaf Mass	Leaf Surface Area	Plant Height	Root Length	Total Biomass	Leaf Mass	Leaf Surface Area	Plant Height	Root Length
T	1.17	55.13 ***	0.02	0.04	4.53 *	1.73	14.13 **	5.53 *	1.03	0.08
C	0.04	0.14	10.3 **	7.12 *	3.11	2.19	42.12 ***	17.07 ***	48.78 ***	7.17 *
T × C	5.95 *	14.98 **	0.49	1.95	0.50	1.33	0.87	1.44	0.04	0.14

Values presented are F-values. Statistically significant differences are marked with asterisks: * *p* < 0.05, ** *p* < 0.01, *** *p* < 0.001, denoting the levels of statistical significance.

**Table 2:** Detailed physiological and biochemical responses of *W. trilobata* and *W. chinensis* to heat wave treatment and culture conditions. This table details the effects of heat wave treatment (T), culture conditions (C), and their interaction (T × C) on chlorophyll content, leaf nitrogen, flavonol content, anthocyanin levels, photosynthetic efficiency (Fv/Fm), net photosynthesis rate (Pn), transpiration rate (Tr), CO_2_ assimilation rate, and water-use efficiency (WUE) in *W. trilobata* and *W. chinensis*.

	*W. trilobata*								
Source	Chlorophyll Content	Leaf Nitrogen	Flavonol	Anthocyanin	Fv/Fm	Pn	Tr	CO_2_	WUE
T	0.03 *	6.51 *	1.77	0.40	48.50 ***	22.48 ***	150.66 ***	50.00 ***	36.14 ***
C	0.18	0.24	14.87 **	0.86	2.48	9.50 **	16.38 ***	3.03	10.56 **
T × C	20.09 ***	22.51 ***	2.79	5.53 *	3.72	0.45	16.38 ***	1.17	0.61
	***W. chinensis***								
**Source**	**Chlorophyll Content**	**Leaf Nitrogen**	**Flavonol**	**Anthocyanin**	**Fv/Fm**	**Pn**	**Tr**	**CO_2_**	**WUE**
T	14.02 **	12.88 **	11.89 **	10.24 **	41.76 ***	28.24 ***	1.29	49.322 ***	28.14 ***
C	8.55 **	7.16 *	14.74 **	7.86 *	3.84	1.00	1.29	49.08 ***	3.36
T × C	7.86 *	7.82 *	0.28	0.24 *	3.85	0.02 *	1.29	49.08 ***	22.40 ***

The data are presented as F-values, with statistically significant differences denoted by *, **, and *** for *p* < 0.05, *p* < 0.01, and *p* < 0.001, respectively.

## Data Availability

The datasets generated during and/or analysed during the current study are available from the corresponding author on reasonable request.
